# Epidemiology of pertussis in Casablanca (Morocco): contribution of conventional and molecular diagnosis tools

**DOI:** 10.1186/s12879-017-2452-3

**Published:** 2017-05-16

**Authors:** Khalid Katfy, Nicole Guiso, Idrissa Diawara, Khalid Zerouali, Bouchra Slaoui, Zineb Jouhadi, Abdelhadi Zineddine, Houria Belabbes, Naima Elmdaghri

**Affiliations:** 1 Laboratoire de Microbiologie, Faculté de Médecine et de Pharmacie, Hassan II University of Casablanca, B, P 5696 Casablanca, Morocco; 2 Service de Microbiologie, CHU Ibn Rochd, B, P 2698 Casablanca, Morocco; 3 Molecular Prevention and Therapy of Human Diseases, Institut Pasteur, 25 rue du Dr Roux, 75015 Paris, France; 4 Service des Maladies Respiratoires Pédiatriques, Hôpital d’Enfants Abderrahim Harouchi, CHU Ibn Rochd de Casablanca, Casablanca, Morocco; 5 Service des Maladies Infectieuses Pédiatriques, Hôpital d’Enfants Abderrahim Harouchi, CHU Ibn Rochd de Casablanca, Casablanca, Morocco; 6 Service d’Accueil des Urgences Pédiatriques, Hôpital d’Enfants Abderrahim Harouchi, CHU Ibn Rochd de Casablanca, Casablanca, Morocco; 7 Bacteriology-Virology and Hospital Hygiene Laboratory, University Hospital Centre Ibn Rochd, 1, Rue des Hôpitaux, 20100 Casablanca, Morocco

**Keywords:** Pertussis, *B. Pertussis*, *B. Parapertussis*, *B. Holmesii*, Pertussis toxin gene, Pertussis whole cell vaccine, RT-PCR

## Abstract

**Background:**

Pertussis, a vaccine preventable disease, is still responsible of significant morbidity and mortality around the world, mostly in newborns. The aim of the present study was (1) to introduce pertussis surveillance in the major pediatric hospital of Casablanca (2) to analyze the prevalence of pertussis among children under 14 years of age and their entourage in Casablanca, Morocco.

**Methods:**

This is a prospective and non-case controlled study, including children suspected of Pertussis admitted at the Abderrahim Harouchi Pediatric Hospital in Casablanca, from January 2013 to June 2015. Nasopharyngeal samples were obtained for *Bordetella spp.* culture and Real time PCR detection (RT-PCR) with specific primers of *Bordetella spp.*, *B. pertussis*, *B. parapertussis* and *B. holmesii*. The detection of *Bordetella spp.* was also performed in some household contacts of the children suspected of pertussis.

**Results:**

During the 2.5-years period, a total of 282 samples were collected from hospitalized children (156) and in some of their contacts (126). Among 156 samples from the children (from whom 57% were under 2 month of age), *Bordetella* DNA was detected in 61% (96/156) by RT-PCR. Among these positive samples, 91.7% (88/96) corresponded to *B. pertussis* DNA. Furthermore, in 39.5% (38/96) of the *Bordetella* positive samples, *B. holmesii* DNA was also detected. *B. parapertussis* DNA was detected in only one sample (1/156). Out of the 156 samples collected from the hospitalized children, only 48 were tested by culture, and 4 *B. pertussis* were isolated (8.3%). Among the 126 samples from the contacts of the children, mostly mothers (115 cases), *Bordetella* DNA was detected in 47% (59/126), 90% (53/59) being *B. pertussis* DNA. Moreover, *B. holmesii* DNA was also detected in 18.6% (11/59) of the *Bordetella* positive samples, and coexistence of *B. pertussis* and *B. holmesii* DNA in 36.5% (35/96). Two *B. pertussis* were isolated by culture performed on 43 samples of the contacts of the children (4.6%).

**Conclusions:**

This study highlights the circulation of *B. pertussis* but also of *B. holmesii* in Casablanca-Morocco with a high proportion of co-infections *B. holmesii/B. pertussis* in infants and their mothers, indicate that infection of non-vaccinated infants could be more associated with young parents**.** Moreover, the RT- PCR provides a sensitive and specific diagnosis of *B. pertussis* infections and distinguishes it from other *Bordetella* species, and is therefore suitable for implementation in the diagnostic laboratory.

## Background

*Bordetella pertussis* is the agent of whooping cough (pertussis), an endemic illness responsible for significant morbidity and mortality, especially in infants and children [1] Worldwide, there are an estimated 16 millions cases of pertussis, 95% of which occur in developing countries, resulting in about 195,000 child deaths per year [1].The epidemiology of pertussis, which is a cyclical disease, is varying from regions to regions because of differences in the type of vaccine used, type of vaccine strategy, and differences in surveillance or lack of surveillance.

Pertussis vaccination has been introduced in almost all vaccination programs throughout the world and has reduced significantly the morbidity and the mortality related to the disease. In the context of high-coverage vaccination coverage, the reported cases are predominantly registered among children < 1 year-old with a high proportion among infants < 3 months-old (Ref). Adults are considered as the main reservoir of transmission of the disease to unvaccinated young children and several immunization strategies have been proposed in order to prevent cases among young children [2].

In Morocco, Pertussis vaccination [pertussis whole cell vaccine (wP) in combination with diphtheria and tetanus toxoids (DTwP)] was introduced by the national immunization program (NIP) in the early 1980’s to prevent pertussis, allowed the reduction of the incidence of the disease from almost 15,000 cases per year in 1980 to less than 100 cases per year by 2011 [3].

Currently, in Morocco, the vaccination strategy includes a primary vaccination at 2, 3 and 4 months of age and two boosters at 18 months of age and 5 years of age. The vaccine is provided through UNICEF. The vaccine pertussis vaccine coverage exceeds 95% at the age of 24 months [4]. Starting in January 2012, a significant increase in the number of cases was registered: during one month, in March 2012, eighty cases were identified and lab-confirmed in the locality of Sale, Morocco. Subsequently, numerous cases were reported across the country, and in August of the same year, another outbreak occurred in Casablanca with more than 130 clinically diagnosed cases hospitalized in the Abderrahim Harouchi children hospital [5].

However, pertussis cannot be confirmed based only on a clinical diagnosis because of the different presentations of the disease in non-vaccinated newborns, partially vaccinated children and adolescents and adults whose immunity waned with time [6].Therefore, laboratory tests are needed for confirmation of the diagnosis in those patients showing suggestive clinical signs and symptoms, or those with a history of exposure to infected patients [7].

Currently available *B. pertussis* diagnostic methods include direct diagnosis such as culture, and nucleic acid amplification assays and indirect diagnosis such as the detection of anti-pertussis toxin antibodies [8]. Culture is specific and sensitive in infants and young children but less in adolescents and adults, coming late after the onset of cough as previously reported by Nakamura et al. 2011 [9]. PCR assays are faster, highly sensitive, and their use in epidemiological surveillance has increased recently to measure the impact of the disease in the community, and to improve studies of vaccine efficacy. However, the routinely real time PCR (RT-PCR) used during the last decade, although very sensitive, is a *Bordetella* PCR. It is based on Insertion Sequence (IS) *481*. This RT-PCR is very sensitive because the IS*481* is present in multicopies on the chromosome of *B. pertussis* [10], however it is no more specific of *B. pertussis* since the target (IS*481*) is also harbored by the chromosome of *B. holmesii* and some *B. bronchiseptica* isolates [11]. *B. holmesii* was first described as the agent of bacteremia in asplenic patients [12]*.* Later on it was first detected in respiratory samples during pertussis outbreaks [13]. It is not yet known whether *B. holmesii* is a real pathogen or an opportunistic bacterium [14]. However, the recent increase of its detection in respiratory samples is mostly due to the use of the RT-PCR based on the IS*481* target as shown in several studies [14–16]. Additional specific RT-PCRs are then needed to determine the nature of the *Bordetella* species responsible of pertussis.

The aim of the present study was (1) to introduce pertussis surveillance in the major pediatric hospital of Casablanca (2) to analyze the prevalence of pertussis among children under 14 years of age and their entourage in Casablanca, Morocco.

## Methods

### Patients

This is a prospective and non-case controlled study including all patients younger than 14 years of age admitted to the Abderrahim Harouchi Pediatric Hospital in Casablanca, Morocco, from January 2013 to June 2015and meet the case definition in accordance with the criteria defined by the WHO [3].

Clinical and epidemiological features of each patient were recorded by a physician, including age, symptoms (coughing, paroxysms, respiratory distress, whoops, apnea, cyanosis, seizure, vomiting), days from the onset of symptoms, and time elapsed until the sample was collected and sent to the laboratory. Surveillance of pertussis has been extended to the household contacts to detect index cases and secondary cases and to prevent the spread of the disease. Duplicates of each sample are excluded from the analysis.

### Samples

Infants (< 12 months) were sampled by nasopharyngeal aspiration using a small catheter inserted through the nostril into the nasopharynx, and flushed with sterile saline. Older children (> 12 months) and adults were sampled by nasopharyngeal swabs which were then placed into a screw cap tube with transport medium (Amies Swab ref. – 300,281 provider DELTALAB).

Nasopharyngeal samples obtained from hospitalized patients, and household contacts were sent to the microbiology laboratory at Ibn Rochd University Hospital in Casablanca at room temperature, accompanied by a fact sheet with all the clinical and socio-demographic indications. All samples were analyzed after an informed consent signed by the parents or caregivers of the children. The research of *Bordetella* was performed also in hospitalized childrens’ contacts after their consent.

### Bacterial culture

The samples were cultured into a fresh Bordet Gengou agar or Regan Low medium (Difco Laboratories, Detroit, USA) prepared in our laboratory, which included 15% of defibrinated horse’s blood and 40 mg/L of cephalexin (Selective supplement *Bordetella*, Oxoïd-Unipath, Dardilly, France). The growth media used for bacterial culture were validated using *B. pertussis* (CIP 8132), *B. parapertussis* (CIP 12822) and *B. holmesii* (CIP104394) reference strains.

They were incubated at 35 ± 2 °C in a moist atmosphere and maintained for three to ten days under aerobic conditions. Colonies were identified based on characteristic morphology, Gram stain, catalase and oxidase testing and by using the API 20NE system (bioMérieux, Marcy L’Etoile, France). The colonies suspected to be *Bordetella spp*. were tested by molecular tools as described below.

### DNA extraction

Nucleic acids were extracted from submitted specimens or bacterial suspensions using a commercial kit (High Pure Template Preparation Kit, Roche Applied Science, Mannheim, Germany) according to the manufacturer’s instructions. The DNA was eluted into a 100-μL volume. The obtained DNA was assayed immediately or stored at −20 °C until use. Concerning the reference strains *B. pertussis CIP* 8132, *B. parapertussis CIP* 12822, and *B. holmesii* CIP 104394, genomic DNA was extracted using DNeasy Tissue Kit (QIAGEN, Hilden, Germany) according to the manufacturer’s protocol.DNA were stored at −20 °C until required for analysis.

### RT- PCR amplification

Detection of *Bordetella* spp. and *Bordetella parapertussis* DNA was performed using Real time PCR (RT-PCR) assays and TaqMan® technology for the amplification of the insertion elements *IS* 481 (*Bordetella* spp), *IS*1001 *(B. parapertussis)*as described by Riffelmann et al. 2005 [10].

Since *IS* 481 target is detecting *B. pertussis* as well as *B. holmesii* DNA, we performed, on all positive *IS* 481 samples, two other specific RT-PCR, one specific of B. pertussis (*ptxA-Pr)* and one specific of B. Holmesii (h-IS*1001*) in order to confirm the diagnosis of *B. pertussis* and/or *B. holmesii* [17, 18]. Negative no-template controls were included in each run. The DNA of *B. pertussis* (Tohama), *B. parapertussis* (12822) and *B. holmesii* (104394) were used as positive controls. DNA was amplified with the CFX96® thermal real-time PCR System (Biorad).Potential inhibitory activity was assessed by amplifying human gene sequences *RnaseP*. A positive result was defined as a cycle threshold (Ct) value below 38 cycles. Ct values of >38 or no detection (Ct value of zero) were considered negative.

### Quality controls

The RT- PCR assays were evaluated through an external quality control program managed by the *Bordetella* National Reference Centre - Institut Pasteur, Paris, France [19].

### Statistical analysis

Data entry was performed using WHONET 5.6 software. Analyzes were performed using Epi info (CDC, Atlanta, Georgia) and Microsoft Excel. Qualitative variable analysis was performed by nonparametric tests: The chi square test or Fisher’s exact test. For quantitative variables the Student’s test (T) was used. A difference is considered statistically significant if *p*-value <0.05.

## Results

During thirty months of surveillance, a total of 282 nasopharyngeal samples were collected from patients with clinical suspicion of pertussis disease and some of their household contacts (156 infants/126 contacts) and were analyzed in the microbiology lab. The majority of patients 85% (132/156 infants) were admitted in the Pediatric Respiratory Diseases ward and 15% (24/156) were hospitalized in intensive care unit indicating the severity of the disease.

The monthly distribution of pertussis cases was not homogeneous. The majority of cases were reported during two periods: 54% (84/156) of cases between April and June, 39% (61/156) of cases between September and November.

Hospitalized children were below 5 years of age, with an average of 60 ± 10 days and 57% (89/156) were under 2 months of age. The average duration of hospitalization was 4 ± 1 days, and for cases hospitalized in intensive care, the average was 28 ± 7 days with the minimum duration to 21 days and the maximum to 35 days according to associated complications. Among the 126 contacts, 115 were children’s mothers; four siblings aged 3 to 6 years and 7 grand-parents. As for the children’s mothers the average age was 26 ± 4.77 years of age.

**Fig. 1:**
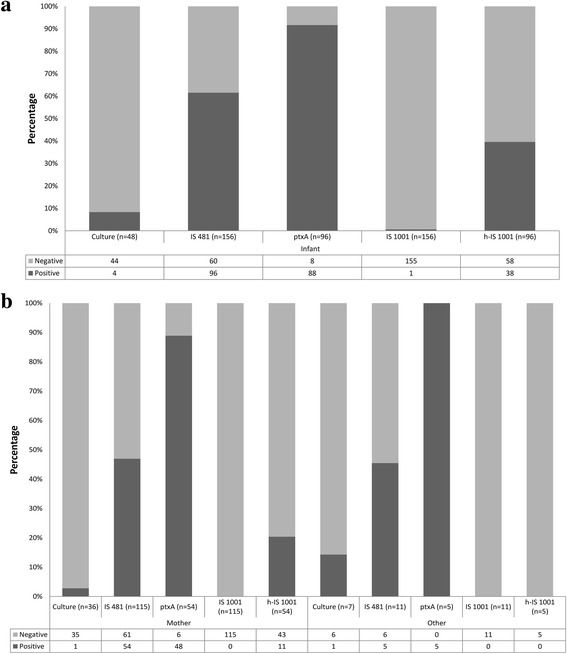
Pertussis confirmed cases by culture and RT-PCR. **a** Confirmed cases among hospitalized patients, **b** Confirmed cases among contact cases

Of the 282 samples collected from all cases, culture was performed only for 91 samples: 48 from hospitalized children and 43 from their contacts. *B. pertussis* was isolated in 8.3% (4/48) and 4.6% (2/43) of the samples, respectively. We collected only 6 *B. pertussis* isolates mainly because most of the time the patients were already treated with macrolides (39/48, i.e. 81.3%) or coming late after the beginning of the cough (27/48, i.e. 56.3%) or because the medium was not available or the laboratory is not working all days at the hospital. All samples were tested by RT-PCR (Fig. 1), *B. parapertussis* DNA was detected in only one sample from hospitalized children. IS*481* RT-PCR was positive in 61.5% (96/156) of the samples from hospitalized children (Fig. 1a). Among these positive samples, *B. pertussis* DNA was specifically detected in 91.7% (88/96) samples, *B. holmesii* DNA was detected in 39.5%, (38/96) and the coexistence of *B. pertussis* and *B. holmesii* DNA being detected in 36.5% (35/96) samples while *B. holmesii* DNA alone was detected only in three samples (Table 1). In 3.1% (3/96) of the *Bordetella* positive samples it was not possible to determine the *Bordetella* species because of the very low amount of DNA*.*

**Table 1:** Results of RT PCR IS*481*, *ptx*-*Pr* and h-IS*1001*

	IS *481*+	IS *481*-	*ptx+/h-IS*1001*+	*ptx*+/h-IS*1001*-	*ptx*-/h-IS*1001*+	*ptx*−/h-IS*1001*-
Patients (*n* = 156)	96	60	35	55	3	3
Contacts (*n* = 126)	59	67	11	42	1	5
Total	155	127	46	97	4	8

*ptx:* pertussis toxin gene*,* h-IS*1001: B. holmesii* specific insertion sequence

IS*481*RT-PCR was positive in 47% (59/126) of the samples from the household contacts. *B. pertussis* DNA was detected in 90% (53/59) of these positive samples, and *B. holmesii* DNA in 18.6% (11/59), always in samples where *B. pertussis* DNA was already detected. In 8.5% (5/59) of the samples it was not possible to determine the *Bordetella* species and they were classified as *Bordetella spp*.

*B. pertussis* and *B. holmesii* DNA were detected in 11 samples from both mothers and siblings. However, *B. holmesii* DNA was also detected in 27 samples from hospitalized children but not in the samples of their mothers. Detection of *B. holmesii* DNA alone was found in one sample of the entourage.

**Fig. 2:**
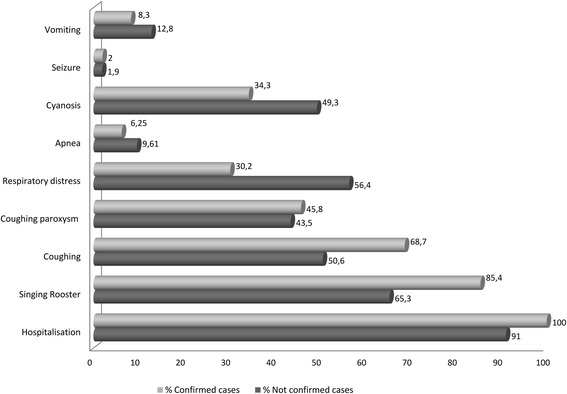
Clinical symptoms of the hospitalized children included in the study

The common symptoms observed among the 96 confirmed patients were whoops 85.4% (82/96), cough 68.7% (66/96) and paroxysmal cough 45.8% (44/96), or cyanosis 34.3%(33/96), whereas apnea 6.25% (6/96) or post-tussive vomiting 8.3% (8/96) presented less frequently (Fig. 2).

Our study showed that *B. pertussis* is also present in healthy people, or with atypical clinical symptoms. Indeed, it has to be noted that 25 confirmed mothers did not declare any clinical symptoms but were in contact to somebody coughing in their household.

It is noteworthy that 89% (78/88) of the confirmed cases had received antibiotic treatment prior to completion of sampling. The administration of Macrolides family antibiotics (Clarithromycin or Azithromycin) was noted in 75% of them, 15% received other antibiotics (cefotaxime, ceftriaxone or cotrimoxazole association) and in 10% of cases, the administered antibiotic was not specified on the information sheet.

The analysis of patients’ vaccination status showed that among the 156 hospitalized children enrolled in the study, 61% (95/156) were unvaccinated and 57% (89/156) were infants less than two months of age and didn’t receive any dose of vaccines according to the Moroccan vaccine strategy recommendation, the other children enrolled 6.3% (6/95) were non vaccinated and were overdue for their first vaccinations between 10 to 30 days. The partially vaccinated patients, 94.2% (49/52) because of their advanced age, they received only 1 to 2 doses. For other 5.8% (3/52) the vaccine recommendations were not followed without medical reasons, and especially for the fourth dose (Table 2).

**Table 2:** PCR results depending on the age and vaccination status

Vaccination status	Age of the patients			
	< 2 months (*n* = 89)		> 2 months (*n* = 67)	
	*B. holmesii*	*B. pertussis*	*B. holmesii*	*B. pertussis*
Vaccinated (*n* = 9)	0	0	3	6
Incompletely vaccinated (*n* = 52)	0	0	9	43
Non vaccinated (*n* = 95)	24	65	2	4
Total	24	65	14	53

During this surveillance, we met children aged 22–38 months who caught whooping cough (*n* = 3) despite they have fulfilled their immunization schedule by vaccinating with 4 doses; other incompletely vaccinated (*n* = 22), with one or two doses were aged between 3 and 14 months.

## Discussion

Following a significant increase of the number of pertussis cases recorded in many parts of the country from January 2012 (Epidemiological Bulletin, June 2012), we decided to establish a laboratory-based surveillance of pertussis, which discriminates among *B. pertussis*, *B. parapertussis* and *B. holmesii*.

During 30 months of surveillance, 282 nasopharyngeal samples were collected from patients with clinical suspicions of pertussis disease (156 infants and126 contacts), were received in our laboratory and were analyzed by RT-PCR and Culture. The majority of patients 85% (132 /156 infants) were admitted in Pediatric Respiratory Diseases service and 15% were hospitalized in intensive care unit indicating the severity of the disease.

Of the 91 samples cultured, 6 (6.5%) were positive for *B. pertussis* by biochemical characters identification, very low yield compared to that obtained by RT-PCR (155/282; 55%).

Culture is the method of choice to isolate the bacteria. However, the chances to obtain positive cultures are high only during the first two weeks of cough and the sensitivity of the method is low as shown in previous studies [7]. In the present study, we collected only 6 isolates for several reasons: (i) 89% were treated by Clarithromycin or Azithromycin therapy before sampling since several days; (ii) most of the contact patients already coughed since 14 days; (iii) sometime the lack of no fresh medium. All these reasons are causes of low sensitivity of the culture as shown in other studies [20, 21]. The treatment of choice in the hospital is macrolides and especially Clarithromycin or Azithromycin.

The direct detection of bacterial DNA by PCR techniques especially RT- PCR is more sensitive than culture (up to 4 weeks after the start of coughing fit) and has the advantage of being faster.

In our study the IS *481* RT-PCR confirmed the circulation of *Bordetella spp.* in the Moroccan population; it infects both unvaccinated infants, teens and adults. To specify *Bordetella* species in the samples, we made complementary RT-PCR such as RT-PCR Ptx-Pr, IS*1001*and h-IS*1001*. The bacterial DNA detected was mostly *B. pertussis* DNA (>90%) whereas *B. parapertussis* DNA was only detected in 0.3% of the samples. The proportion of *B. parapertussis* cases is much lower than in Tunisia where whooping cough was attributed to *B. parapertussis* in 7% of cases [22].

RT-PCR h-IS*1001* detected *B. holmesii* DNA in several samples of hospitalized infants (38/96).Surprisingly the large majority of the *B. holmesii* positive samples were also *B. pertussis* positive samples (35/96) indicating co-infection with both bacteria. Furthermore, both bacteria were detected in samples of 11 infants and their mothers suggesting transmission between the mother and the infant but in 27infant cases, the mothers were only infected by *B. pertussis* indicating that the mothers were not the contaminator of *B. holmesii*. The contaminator could have been another member of the household or another reservoir still not known such as animals or the environment. It was already described that the prevalence of *B. holmesii* can widely differ from one region to another even if the regions are geographically close [14] and detected in all age group. This carriage of *B. holmesii* in infants is different from what was observed in France, Tunisia or US [15, 16, 22] but similar to what was found in Chile, Romania and Argentina [23–25]. In our study, we identified three patients infected with only *B. holmesii*; two children lived in slums and the third lived in an orphanage. This leads us to think that the origin of the infection can be animals or environment. Similarly, these children had forms of immunodeficiency (asplenia, chronic obstructive pulmonary disease), they had clinical symptoms compatible with whooping cough but the search for viral coinfection or mycoplasma was not searched.

Misdiagnosis of *B. holmesii* respiratory infection as *B. pertussis* can lead to wrong diagnosis and this affects epidemiological analysis. For this reason it is important to use specific diagnosis even if a little bit less sensitive.In our study there was a high prevalence of co-infections with *B. pertussis*/*B. holmesii,* as observed in Romania [25]. It is not the case in all recent studies. For example there was no detection in Tunisia [22], detection mainly in adults and in adolescents in France but no co-infection [15] and in US with co-infection or not [16], detection in children in Chile and Argentina [23, 24]. The isolation or detection of *B. holmesii* is recent and deserves further studies, in particular on the clinical symptoms induced by this *Bordetella* species. It is difficult in our study to describe *B. holmesii* clinical symptoms since in most of the cases we observed co-infection with *B. pertussis*. *B. holmesii* DNA alone was detected only in three cases but the search for viral or mycoplasma infection was not searched and the clinical symptoms induced cannot be linked to *B. holmesii* only.

Surveillance and awareness of this species need to be pursued in order to understand its epidemiology [26].

It should be noted that the main objective of vaccine is not only to prevent pertussis in adults but to avoid the contamination of infants too young to be vaccinated by their relatives.

This paradoxical resurgence of whooping cough is not due to solely a progressive loss of immunity in the time or a decrease in immunization coverage in some regions (deletion recall or non-compliance with recommendations), but also be due to lack of vigilance of parents who often slow to vaccinate their children on time, or introducing a new more sensitive laboratory diagnosis such as RT-PCR.

Also, instead of thinking that to prevent infection in early childhood, through the application of booster vaccinations with their entourages (adolescent or adults), we should seek to achieve an early and timely vaccination. WHO propose to initiate at 6 weeks and no later than 8 weeks of age, and maintain high coverage (≥90%) with at least 3 doses [27].

## Conclusion

We have highlighted the circulation of *Bordetella* in patients hospitalized at the pediatric hospital by rapid diagnosis based mostly on RT- PCR. The results obtained within this study encourage us to expand it to other laboratories in Morocco in order to improve the biological diagnostic and surveillance of bordetelloses.

In fact we found a high proportion of *B. pertussis* and *B. holmesii* co-infection, it is then important to continue the surveillance in order to have a better understanding of the *Bordetella spp*. circulating in Moroccan population and their role in the respiratory symptoms.

Moreover, our study suggests that infection of non-vaccinated infants is associated with parents, hence the need to investigate around the index cases to detect contaminators and prevent contamination of young infants and disease spread by quick treatment. Furthermore, the RT- PCR provides a sensitive and specific diagnosis of *B. pertussis* infections and distinguishes it from other *Bordetella* species, and is therefore suitable for implementation in the diagnostic laboratory.

## Abbreviations

*ptx*: pertussis toxin gene
RT-PCR: real time polymerase chain reaction
WHO: World Health Organization
